# Daily consumption of γ-oryzanol-fortified canola oil, compared with unfortified canola and sunflower oils, resulted in a better improvement of certain cardiometabolic biomarkers of adult subjects with type 2 diabetes: a randomized controlled clinical trial

**DOI:** 10.1186/s40001-023-01409-8

**Published:** 2023-10-10

**Authors:** Bahareh Nikooyeh, Azizollaah Zargaraan, Samira Ebrahimof, Ali Kalayi, Maliheh Zahedirad, Hootan Yazdani, Marjan Rismanchi, Taher Karami, Marzieh Khazraei, Ali Jafarpour, Tirang R. Neyestani

**Affiliations:** 1 Laboratory of Nutrition Research, National Nutrition and Food Technology Research Institute and Faculty of Nutrition Sciences and Food Technology, Shahid Beheshti University of Medical Sciences, Tehran, Iran; 2 Department of Food and Nutrition Policy and Planning Research, National Nutrition and Food Technology Research Institute and Faculty of Nutrition and Food Science, Shahid Beheshti University of Medical Sciences and Health Services, Tehran, Iran; 3 Department of Research and Development, Kourosh Food Industry, Tehran, Iran; 4 Quality Assurance Unit, Kourosh Food Industry, Tehran, Iran

**Keywords:** Type 2 diabetes, γ-oryzanol, Canola oil, Sunflower oil, Clinical trial

## Abstract

**Background:**

This study was undertaken to examine the effects of daily consumption of γ-oryzanol (ORZ)-fortified canola oil, as compared with plain canola and sunflower oils, on certain cardiometabolic indicators.

**Methods:**

Ninety-two adult subjects from both sexes with T2D were randomly assigned to one of the three groups to receive: (a) ORZ-fortified canola oil (Group 1; n_1_ = 30); (b) unfortified canola oil (Group 2; n_2_ = 32); or (c) sunflower oil (Group 3; n_3_ = 30) for 12 weeks. The participants were instructed to use only the given oils for all cooking (but frying) purposes. Anthropometric, dietary and biochemical assessments were done initially and finally.

**Results:**

Though body mass index (BMI) significantly decreased in all three groups, only in Groups 1 and 2 waist circumference (WC) showed a significant decrement (-2.6 ± 0.1 and -2.2 ± 0.1 cm in Groups 1 and 2 respectively, p < 0.001 for both) which was accompanied by a significant reduction of blood pressure just in Group 1. Fasting blood glucose (FBG) and glycated hemoglobin (HbA1c) showed a significant decrease only in ORZ-fortified canola oil group (−7.7 ± 0.4 mg/dL, p = 0.039 and −0.7 ± 0.1%, p < 0.001, respectively). However, insulin resistance, as judged by HOMA-IR, did not change significantly. In addition, serum triglyceride (TG) concentrations decreased in all three groups but only in ORZ-fortified canola oil was this decrement statistically significant (-17.9 ± 2.1 mg/dL, p = 0.005). Other components of serum lipid profile did not change significantly in either group.

**Conclusions:**

Consumption of either sunflower or canola oils for 12 weeks improved certain studied biomarkers. However, only ORZ-fortified canola oil resulted in a significant decrease of blood pressure, WC, FBG, HbA1c and TG. These findings can help both clinicians and public health authorities for dietary recommendations to subjects with T2D and presumably the whole community.

*Trial registration*: number at clinicaltrials.gov (NCT05271045).

## Introduction

Cardiovascular disease (CVD) is the most prevalent morbidity and the main cause of mortality in subjects with type 2 diabetes (T2D). It has been estimated that CVD may develop in about 32% of all subjects with T2D with coronary artery disease (CAD) and cerebrovascular accident (CVA) as the main causes of death [1]. Some studies indicate that CVD death in the context of T2D is more prevalent in low and middle income communities [2]. Though persistent high blood glucose is definitely a contributor to CVD risk, there may also be some other predisposing factors like deranged blood lipids [3]. Cardiometabolic risk factors including abdominal adiposity, dyslipidemia and high blood pressure all contribute to development of diabetes and its further complications [4]. Fortunately, most of these are modifiable and that is why having a healthy diet and life style is the main step in prevention and treatment of T2D [5, 6].

Dietary fats, as one of the main dietary components, may affect most, if not all, cardiometabolic risk factors including body weight [7, 8], blood glucose [9, 10] and lipids [11, 12]. A huge body of evidence indicates that replacement of saturated fats with liquid plant oils containing mono- and polyunsaturated fatty acids (MUFAs and PUFAs, respectively) may be accompanied by reduced risk of CVD [13], the primary cause of mortality in T2D [2].

Healthy plant oils may contain phytochemicals, naturally occurring bioactive compounds carrying various health benefits including antidiabetic effects [14]. One of the phytochemicals recently received huge attention is γ-oryzanol (ORZ), a phytochemical of rice bran oil. An increasing body of evidence indicates the beneficial effects of ORZ on cardiometabolic risk factors including increased body weight/fat [15, 16], deranged blood lipids [17, 18], raised blood pressure [19] and dysglycemia [18, 20]. As a result, ORZ has been considered as an adjunct approach to the currently recommended life style modifications to prevent and treat T2D and its complications [21–23]. Notwithstanding, the effects of daily intake of ORZ under free living conditions with no predetermined intake dose on cardiometabolic biomarkers in T2D have not been evaluated to date.

This study was undertaken to examine the effects of daily consumption of ORZ-fortified canola oil, as compared with plain canola and sunflower oils, on certain cardiometabolic biomarkers. We chose canola oil as a vehicle for ORZ fortification because: (i) oils as staple foods in Iran are suitable for fortification purposes [24, 25], and (ii) canola oil containing 60% oleic acid (C18:1), 20% linolenic acid (C18:2) and 10% α-linolenic acid (C18:3) is one of the healthiest oils [26]. We used sunflower oil to compare the effects of ORZ-fortified and –unfortified canola oils with because (i) it is the most common liquid plant oil used in Iran; and (ii) having about 85% unsaturated fatty acids including linoleic and oleic acids, it is also considered a healthy oil [27].

As most liquid cooking oils available in the Iranian market are voluntarily fortified with vitamins A and D, all oils used in this study contained equal amounts of these two vitamins. Nevertheless, we refer to the canola oil without ORZ as “unfortified” throughout this article just for convenience.

## Materials and methods

### Study design

**Fig. 1:**
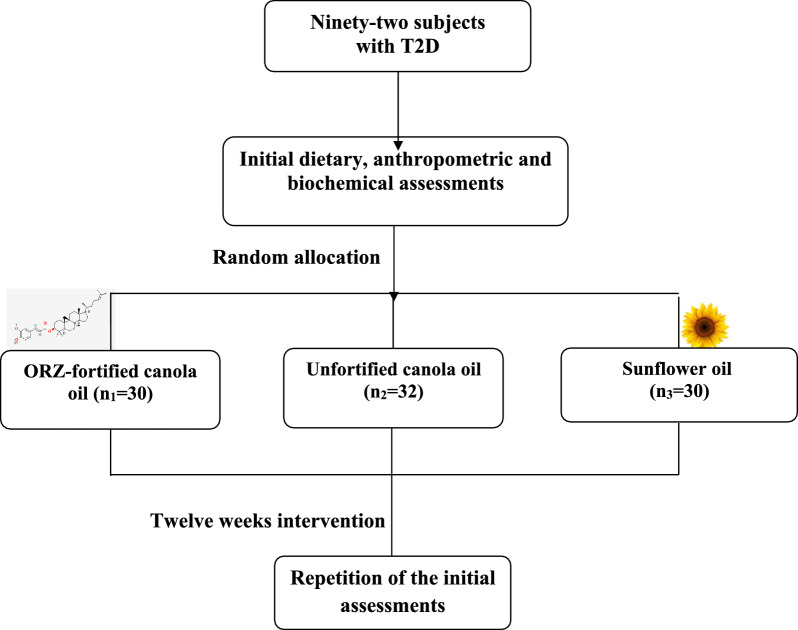
The study protocol at a glance

The study protocol has been comprehensively described elsewhere [28]. This was a double-blind clinical trial. Both the subjects and the research team, except for two main supervisors, were unaware of the group allocations. Briefly, adult subjects with T2D were recruited from Iran Diabetes Society and general population using announcements. The protocol and objectives of the study were fully described to those who met the inclusion criteria and were willing to participate in the study before they signed an informed consent. The inclusion criteria were (i) confirmed diagnosis of T2D; (ii) age 20–65 years; (iii) not receiving insulin. The participants were randomly assigned to one of the three groups to receive: (a) ORZ-fortified canola oil (1 mg ORZ/1 g; Group 1); (b) unfortified canola oil (without ORZ; Group 2); or (c) sunflower oil (Group 3). We employed the block randomization method taking into account the participants’ sex and age. This method is an effective approach for mitigating bias in the allocation process when implemented correctly [29]. The participants were instructed to use only the given oils for all cooking (but frying) purposes. Enough oils were given to the participants for their whole household consumption (based on 30 g/person) on a monthly basis. At the end of each month, the participants were bringing the remainder of the unused oils with them to our lab to calculate the amount of consumed oil and then new packs of oils were given to them. The duration of intervention was 12 weeks. Those subjects with any significant change in diet, life style or medications and those who had eaten out for three consecutive days or more than ten days during the intervention period would be excluded. All assessments were performed at the beginning and in the end of the intervention for all subjects. Figure 1 demonstrates the study protocol schematically.

### Assessments

#### Dietary intake

To assess dietary intake, a 24 h dietary recall questionnaire was completed for two days, including one holiday by an experienced nutritionist. Dietary data were converted to energy and nutrients using Nutritionist IV (version 4.1, 1997; First DataBank, The Hearst Corporation, San Bruno, CA).

#### Anthropometric and blood pressure measurements

Weight and height were measured using standard methods to the nearest of 0.1 kg and 0.1 cm, respectively. Body mass index (BMI) was calculated using weight (kg)/height (m)^2^. Waist circumference (WC) was measured using a measuring tape to the nearest of 0.1 cm.

Blood pressure was measured while the subject was in a sitting position and after at least five minutes rest. In this study, the mean of two measurements was considered as the subject’s blood pressure value (mmHg) [28].

#### Laboratory evaluations

##### Collecting and handling of specimens

Venous blood sample was taken from all participants following an overnight fasting (12–14 h). Sera recovered from clotted samples following centrifugation at 800 g at room temperature were aliquoted in several fresh microtubes. One aliquot was used for serum glucose and lipid profile determination on the same day of sampling whereas the other microtubes were kept at −80° C until the day of analysis.

##### Biochemical measurements

Concentrations of fasting serum glucose (FSG), triglycerides (TG), total cholesterol (TC), high-density lipoprotein cholesterol (HDL-C) and low-density lipoprotein cholesterol (LDL-C) were determined by enzymatic methods (all from Pars-Azmoon, Tehran, Iran) using an auto-analyzer (Selecta E; Vitalab, Holliston, Netherlands). Glycated hemoglobin (HbA1c) was evaluated using enzymatic method (Pishtaz Teb, Tehran, Iran). Insulin resistance was evaluated using homeostasis model assessment-estimated insulin resistance (HOMA-IR) [30].

## Statistical analyses

Normal distribution of the data was evaluated using Shapiro–Wilk’s test. Descriptive and qualitative data were presented as mean ± standard deviation (SD) and number (percent), respectively. Paired *t* test and Wilcoxon were employed for within-group comparisons of data with and without normal distribution, respectively. Analysis of covariate (ANCOVA) with adjustment for basal (time zero) variables was used for between-group comparisons. In this study, p < 0.05 was considered statistically significant. All analyses were performed using Statistical Software for Data Science (STATA, version 17, StataCorp LLC, Texas, US).

## Ethical issues

The protocol and objectives of the study were clearly explained for all participants before they signed a written informed consent. The protocol of this study was approved by the Ethics Committee of National Nutrition and Food Technology Research Institute (NNFTRI). This clinical trial was registered at clinicaltrials.gov (NCT05271045).

## Results

Ninety-two subjects from both sexes aged 57.6 ± 1.9 y completed the study. There were no significant between-group differences in demographic variables or the amount of consumed oil per capita (Table 1). There were no complaints of the organoleptic properties of the oils and nor was there any attrition throughout the intervention period indicating that the compliance was 100%. Dietary intake data showed no statistically significant inter- or intra-group differences (Table 2).

**Table 1:** Age, sex and per capita oil consumption of the studied groups

Variable	Group 1 (n_1_ = 30)	Group 2 (n_2_ = 32)	Group 3 (n_3_ = 30)	p value
Age (y)	53.9 ± 1.9	58.7 ± 1.8	60.1 ± 1.9	0.064
Sex: No (%)				
Male	17 (56.7)	20 (62.5)	17 (56.7)	0.864
Female	13 (43.3)	12 (37.5)	13 (43.3)	
Per capita oil consumption (g/d)*	29.1 ± 0.7	28.7 ± 0.8	30.2 ± 0.4	0.367

Group 1: γ-oryzanol-fortified canola oil; Group 2: Unfortified canola oil (without γ-oryzanol); Group 3: Sunflower oil

^*^The amounts of consumed oil denotes the amount of the oils given to the participants and not total fat intake

**Table 2:** Intra- and inter-group comparisons of mean daily energy and some selected nutrients intakes

Variable	Group 1 (n_1_ = 30)			Group 2 (n_2_ = 32)			Group 3 (n_3_ = 30)			P_2_	P_3_	P_4_
	Initial	Final	P_1_	Initial	Final	P_1_	Initial	Final	P_1_			
Energy (kcal)	1682.2 ± 99.6	1738.8 ± 126.4	0.610	1662.4 ± 63.0	1801.3 ± 98.2	0.130	1718.6 ± 65.1	1695.7 ± 107.0	0.838	0.828	0.680	0.357
Carbohydrate (g)	246.9 ± 20.3	306.4 ± 57.7	0.263	255.4 ± 13.5	271.3 ± 18.2	0.271	261.2 ± 11.1	259.5 ± 17.0	0.926	0.624	0.461	0.947
Protein (g)	64.1 ± 3.2	64.4 ± 5.0	0.953	62.6 ± 3.0	70.0 ± 4.8	0.139	61.1 ± 3.2	60.2 ± 3.7	0.405	0.579	0.661	0.182
Fat (g)	51.6 ± 3.4	50.4 ± 4.0	0.775	46.2 ± 3.2	46.9 ± 3.3	0.820	48.8 ± 2.9	45.7 ± 3.5	0.311	0.995	0.726	0.752
Total dietary fiber (g)	15.2 ± 1.2	14.2 ± 1.3	0.381	15.3 ± 0.8	16.3 ± 1.2	0.514	12.7 ± 1.2	14.6 ± 2.0	0.440	0.588	0.828	0.939
Vitamin A (μg)	753.5 = 131.2	573.0 ± 115.9	0.272	569.6 ± 97.0	419.5 ± 41.5	0.174	807.4 ± 198.4	519.6 ± 72.3	0.159	0.452	0.883	0.779
Iron (mg)	13.8 ± 1.0	13.7 ± 1.1	0.983	14.3 ± 0.6	16.1 ± 1.4	0.242	13.2 ± 0.7	14.9 = 2.5	0.311	0.622	0.735	0.991
Zinc (mg)	6.4 ± 0.4	5.8 ± 0.4	0.315	5.7 ± 0.3	6.5 ± 0.8	0.349	6.6 ± 0.3	5.5 ± 0.7	0.153	0.509	0.907	0.816
Calcium (mg)	572.0 ± 40.2	564.3 ± 44.4	0.869	576.4 ± 43.3	591.4 ± 41.0	0.748	574.7 ± 37.8	571.6 ± 50.9	0.562	0.902	0.531	0.303

Group 1: γ-oryzanol-fortified canola oil; Group 2: Unfortified canola oil (without γ-oryzanol); Group 3: Sunflower oil

P_1_: Intra-group comparison; P_2_: Comparison between groups 1 and 2; P_3_: Comparison between groups 1 and 3; P_4_: Comparison between groups 2 and 3

Table 3 demonstrates changes of the studied cardiometabolic markers in the three groups. Intra-group changes of BMI showed a significant decrease in all three groups and there was no significant inter-group difference. Notwithstanding, WC decreased significantly only in two canola consuming groups and the changes were statistically significant as compared with group 3 (sunflower oil). Interestingly, both systolic and diastolic blood pressure (SBP and DBP, respectively) reduced significantly just in Group 1. In between-group comparisons, SBP changes were significantly different as compared with group 3 (p = 0.049) and this difference remained statistically significant after adjustment for final values of BMI (p = 0.049) but disappeared after adjustment for WC final values (p = 0.076).

**Table 3:** Intra- and inter-group comparisons of anthropometric, blood pressure and biochemical biomarkers

Variable	Group 1 (n_1_ = 30)			Group 2 (n_2_ = 32)			Group 3 (n_3_ = 30)			P_2_	P_3_	P_4_
	Initial	Final	P_1_	Initial	Final	P_1_	Initial	Final	P_1_			
BMI (kg/m^2^)	28.4 ± 0.79	27.9 ± 0.77	< 0.001	29.7 ± 0.72	28.3 ± 0.71	< 0.001	29.2 ± 0.68	28.5 ± 0.72	0.019	0.831	0.753	0.390
WC (cm)	99.7 ± 2.0	97.03 ± 2.0	< 0.001	104.6 ± 1.9	102.4 ± 1.9	< 0.001	102.7 ± 1.4	102.2 ± 1.4	0.472	0.894	0.017	0.047
SBP (mmHg)	135.5 ± 2.6	127.9 ± 2.7	0.035	139.6 ± 2.9	134.0 ± 2.4	0.078	141.9 ± 3.8	140.8 ± 4.3	0.661	0.581	0.049	0.306
DBP (mmHg)	84.7 ± 1.6	76.3 ± 3.0	0.0175	85.6 ± 1.4	83.9 ± 1.6	0.266	86.3 ± 1.5	84.4 ± 2.0	0.315	0.057	0.059	0.997
FBG (mg/dL)	134.0 ± 6.2	126.4 ± 5.8	0.039	157.1 ± 13.8	141.2 ± 11.1	0.090	144.6 ± 8.1	152.4 ± 11.6	0.367	0.990	0.157	0.113
HbA1c (%)	6.1 ± 0.12	5.4 ± 0.12	< 0.001	6.1 ± 0.24	5.8 ± 0.20	0.053	5.9 ± 0.18	6.0 ± 0.20	0.472	0.010	< 0.001	0.093
Insulin (μIU/mL)	17.2 ± 2.2	16.2 ± 1.9	0.437	16.6 ± 2.1	16.5 ± 1.8	0.942	16.9 ± 2.2	18.3 ± 2.2	0.343	0.893	0.380	0.640
HOMA-IR	5.6 ± 0.7	4.9 ± 0.6	0.168	5.7 ± 0.7	5.5 ± 0.6	0.239	5.7 ± 0.8	6.5 ± 0.8	0.228	0.976	0.079	0.118
TG (mg/dL)	134.7 ± 9.8	116.8 ± 7.8	0.005	147.1 ± 11.8	129.8 ± 10.5	0.141	131.1 ± 10.5	115.7 ± 7.7	0.070	0.776	0.997	0.815
TC (mg/dL)	145.1 ± 5.5	142.8 ± 6.9	0.535	162.2 ± 7.3	159.8 ± 7.7	0.592	150.6 ± 5.6	149.0 ± 6.8	0.785	0.949	0.976	0.994
HDL-C (mg/dL)	58.9 ± 2.8	58.3 ± 2.8	0.795	59.1 ± 3.3	57.0 ± 2.5	0.489	62.5 ± 2.4	58.7 ± 2.4	0.156	0.894	0.947	0.991
LDL-C (mg/dL)	100.3 ± 3.6	100.9 ± 4.1	0.850	109.3 ± 6.4	105.3 ± 6.7	0.388	100.2 ± 5.1	102.9 ± 5.9	0.597	0.919	0.937	0.740

Group 1: γ-oryzanol-fortified canola oil; Group 2: Unfortified canola oil (without γ-oryzanol); Group 3: Sunflower oil

P_1_: Intra-group comparison; P_2_: Comparison between groups 1 and 2; P_3_: Comparison between groups 1 and 3; P_4_: Comparison between groups 2 and 3

*BMI* body mass index, *DBP* diastolic blood pressure, *FBG* fasting blood glucose, *HbA1c* glycated hemoglobin, *HDL-C* high-density lipoprotein cholesterol, *HOMA-IR* homeostatic Model Assessment for Insulin Resistance, *LDL-C* low-density lipoprotein cholesterol, *SBP* systolic blood pressure, *TC* total cholesterol, *TG* triglycerides, *WC* waist circumference

FSG concentrations decreased in both Groups 1 and 2 but only in Group 1 was the decrement statistically significant. In Group 3, there was an insignificant increase in FSG. Similarly, HbA1c showed a decrease in both canola consuming groups which was significant in Group 1 (6.1 ± 0.12 vs. 5.4 ± 0.12% p < 0.001) and close to significant in group 2 (6.1 ± 0.24 vs. 5.8 ± 0.20%, p = 0.053). HbA1c did not change in Group 3. Other biomarkers of glycemic status including fasting serum insulin and HOMA-IR did not show any significant intra- or inter-group difference. Among the components of serum lipid profile, only serum TG showed a reduction in all three groups which was statistically significant just in Group 1 (134.7 ± 9.8 vs. 116.8 ± 7.8 mg/dL, p = 0.005).

## Discussion

We found a significant decrease in BMI following 12 weeks intervention in all three groups. Though consumption of canola oil may bring about a slight weight reduction [31], it is plausible that this weight reduction have been resulted from unconsciously paying more attention to the amount of dietary, including oil, intake in all three groups. To support this notion, dietary intake data did not show any significant intra- or inter-group changes. Significant decrement of WC in just two canola-consuming groups deserves great attention. The effect of canola oil consumption on WC has already been reported and has been suggested to be due to its oleic acid content [32]. Nevertheless, addition of ORZ did not potentiate the effect of canola oil on WC.

The effect of ORZ-fortified canola oil consumption on blood pressure is noteworthy. Some evidence indicates the reducing effect of canola oil on blood pressure through reduction of visceral fat [32]. However, absence of any significant change in blood pressure in Group 2 suggests the effect of added ORZ. In support of this notion, a clinical trial failed to show any significant change in blood pressure of adult subjects with T2D who consumed 30 g/d canola oil for eight weeks [33]. Removal of significant difference of SBP between groups 1 and 3 in the current study indicates that the effect of ORZ on SBP is, at least in part, mediated through reduction of WC and independent of BMI. Along the same line, in a population-based study a direct relationship between blood pressure and WC, independent of BMI, was observed [34]. Current evidence on the effect of ORZ on blood pressure is scarce. In an experimental study, the reducing effect of rice bran extract (RBE) on blood pressure of hypertensive rats was documented [35]. Notwithstanding, this effect might have been due to ferulic acid in RBE, that may decrease blood pressure via induction of nitric oxide (NO) generation in blood vessels [36]. It is worth to note that the majority of our participants were normotensive and hence might be less responsive to the effect of ORZ on blood pressure. The effect of ORZ on blood pressure deserves further studies.

Twelve weeks consumption of ORZ-fortified canola oil resulted in a significant decrease in both FBG and HbA1c in adult subjects with T2D. However, insulin resistance, as judged by HOMA-IR, did not change significantly. Though similar changes were observed in unfortified canola oil group, they were not statistically significant. Studies on the effect of canola oil on glycemic indicators are scarce. A clinical trial on the effects of eight weeks consumption of 30 g/d canola or olive oil, as compared with sunflower oil, failed to show any significant improvement in FBG and serum insulin concentrations in adult subjects with T2D [33]. A meta-analytical study also reported almost similar findings [31]. In contrast, in a study reported from India, six moths consumption of canola oil resulted in a significant decrease in FBG in adult subjects with non-alcoholic fatty liver (NAFLD) [37]. Canola oil is a rich source of MUFAs, whose ameliorating effect on HbA1c has been demonstrated by a meta-analysis study [38]. Altogether, it is likely that duration of consumption is a crucial determinant of the effect of canola oil on glycemic status. It is noteworthy that both canola-consuming groups had a significant decrease in BMI. A recent meta-analysis study reported the effect of canola oil consumption on weight reduction [31]. It is, therefore, possible that the improvement of glycemic biomarkers had been due to decrement of BMI. It has been estimated that for each kilogram weight loss, there may be 0.1% decrease in HbA1c [39]. In the current study, both canola-consuming groups (groups 1 and 2) had almost 1.2 kg weight loss, which was accompanied by about 10% reduction in HbA1c. This reduction was significantly more in Group 1 than in group 2 indicating the synergistic and weight-independent effect of ORZ and canola oil on HbA1c.

Serum TG concentrations in all three groups decreased but only in group 1 (canola oil + ORZ) was this decrement statistically significant. Other components of serum lipid profile did not change significantly in either group. Contrary to our finding, improvement of serum lipid profile was observed in a large multi-center clinical trial [40]. Though serum lipid profile, notably TG concentrations, may improve following weight loss [41], since there was a similar weight loss in both canola consuming groups, reduction of serum TG concentrations only in Group 1 was likely due to the added ORZ. The effect of ORZ intake on serum TG concentrations has already been demonstrated in animal model [20]. Notwithstanding, in a controlled clinical trial on dyslipidemic subjects, consumption of rice bran oil containing ORZ for four weeks did not significantly affect lipid profile components but serum LDL-C concentration [17]. Experimental studies indicate that ORZ, through inhibition of stearyl CoA saturase, impedes TG accumulation in the liver [42].

To the best of our knowledge, this is the first clinical trial of the effectiveness of daily consumption of ORZ-fortified canola oil, as compared with unfortified canola and sunflower oils, on anthropometric, glycemic and lipidemic biomarkers of the adult subjects with T2D. It is noteworthy that in this study, unlike similar works, there was no predetermined amount of the oils to be consumed. Nevertheless, some limitations are acknowledged. Our dietary intake assessment using 24 h dietary recall for two days failed to show any changes despite significant reduction of BMI in the three studied groups. It has been recommended to repeat 24 h recall for 4 to eight days to show the variations [43]. Nevertheless, several repetition of 24 h recall might limit the participation and resulting in selection bias [44]. In addition, having a control group with no intervention would help better interpretation of the findings. However, blinding for a group of subjects with no intervention would be impossible.

In conclusion, replacing common cooking oils, including animal oils, with either sunflower or canola oils for 12 weeks improved certain studied biomarkers notably BMI, WC and serum TG concentrations. However, only ORZ-fortified canola oil resulted in a significant decrease of blood pressure and glycemic biomarkers, notably FBG and HbA1c. These findings can help both clinicians and public health authorities for dietary recommendations to subjects with T2D and presumably the whole community.

## Data Availability

All data will be available upon a reasonable request to the corresponding author.

## Abbreviations

ANCOVA: Analysis of covariate
BMI: Body mass index
CAD: Coronary artery disease
CVA: Cerebrovascular accident
CVD: Cardiovascular disease
DBP: Diastolic blood pressure
FSG: Fasting serum glucose
HbA1c: Glycated hemoglobin
HDL-C: High-density lipoprotein cholesterol
HOMA-IR: Homeostasis model assessment-estimated insulin resistance
LDL-C: Low-density lipoprotein cholesterol
MUFAs: Mono-unsaturated fatty acids
NAFLD: Non-alcoholic fatty liver disease
NNFTRI: National Nutrition and Food Technology Research Institute
NO: Nitric oxide
ORZ: Gamma-oryzanol
PUFAs: Poly-unsaturated fatty acids
RBE: Rice bran extract
SBP: Systolic blood pressure
SD: Standard deviation
TC: Total cholesterol
T2D: Type 2 diabetes
TG: Triglyceride
WC: Waist circumference
